# Supplementary material for: OS-PCA: Orthogonal Smoothed Principal Component Analysis Applied to Metabolome Data
Source: Metabolites. 2021 Mar 5;11(3):149. doi: 10.3390/metabo11030149 (PMC7999099; doi:10.3390/metabo11030149)
Supplement: Supplementary file 1 [file metabolites-11-00149-s001.pdf]

Supplementary Materials

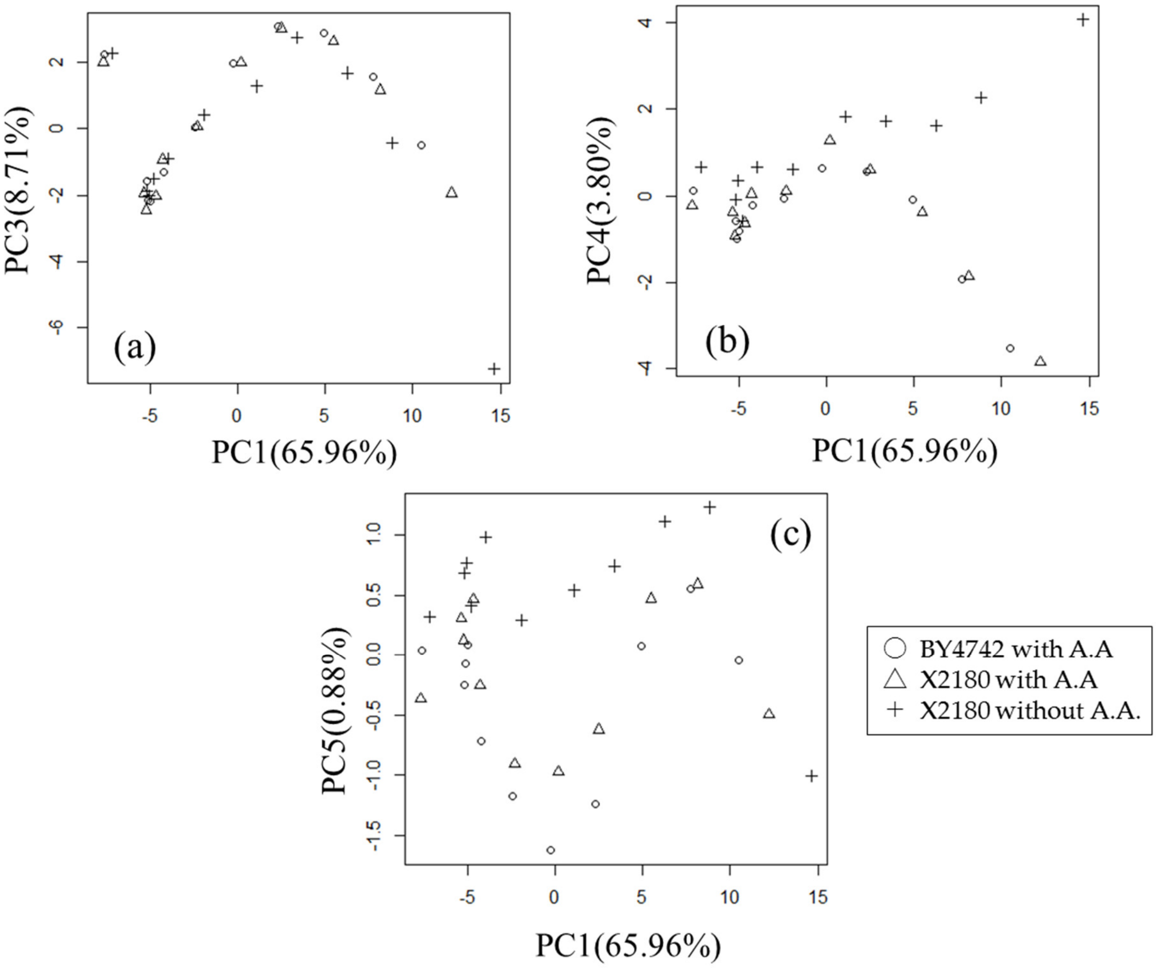

Figure S1. Scatter plot of PC scores obtained by PCA of the metabolic turnover data of Nakayama et al. [15]. (a) Scatter plot of first and third PC scores (PC1 and PC3). The contribution ratio (variance) of PC3 was 16.76%. (b) Scatter plot of first and fourth PC scores (PC1 and PC4). The contribution ratio (variance) of PC4 was 3.80%. (c) Scatter plot of first and fifth PC scores (PC1 and PC5). The contribution ratio (variance) of PC5 was 0.88%. (○) *S. cerevisiae* BY4742 cultured in SD medium with amino acids (A.A.), (△) *S. cerevisiae* X2180 cultured in SD medium with amino acids, (+) *S. cerevisiae* X2180 cultured in SD medium without amino acids.

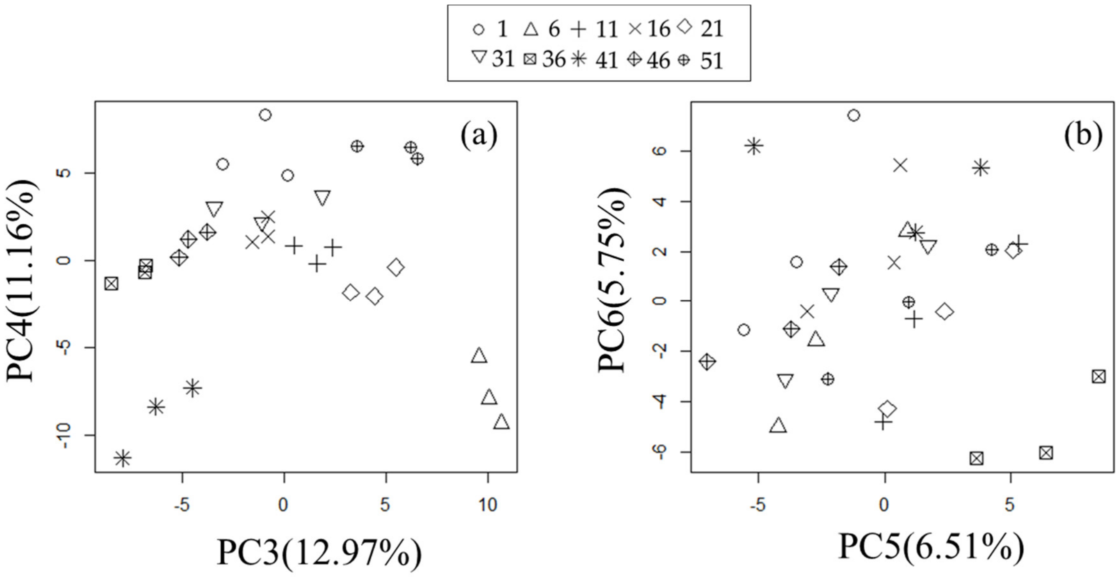

Figure S2. Scatter plots of PC scores obtained by PCA of the metabolome data for taste testing of Japanese green tea. (a) Scatter plot of third and fourth PC scores (PC3 and PC4). The contribution ratios (variance) of PC3 and PC4 were 12.97% and 11.16%, respectively. (b) Scatter plot of fifth and sixth PC scores (PC5 and PC6) were 6.51% and 5.75%, respectively. The tea leaf ranks were (○) 1, (△) 6, (+) 11, (×) 16, (◇) 21, (▽) 31, (⊠) 36, (✳) 41 (⟐) 46, (⊕) 51.

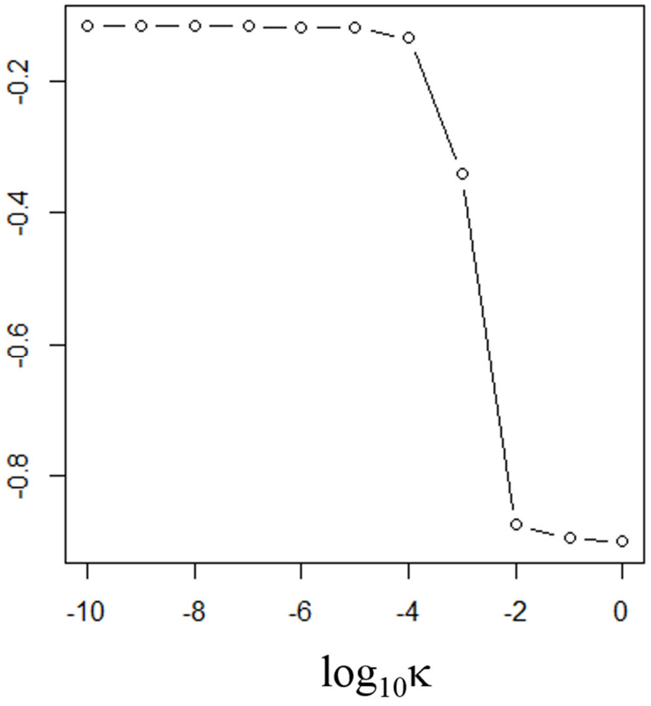

Figure S3. Correlation coefficient plot between the OS-PC1 score and dummy variable of taste ranking of Japanese green tea. The horizontal axis indicates the $\log_{10}$ value of κ from 10^{-10} to 0.999 and the vertical axis indicates correlation coefficient between the OS-PC1 score and a dummy variable of the taste ranking.

Table S1. Statistically significant metabolites correlated with the OS-PC2 score obtained by OS-PCA of metabolic turnover data of Nakayama et al. [7].

Peak	Annotaton	w_x	R	*p*-value		*q*-value	
Peak-01		0.0673	-0.0931	0.6063		0.9765	
Peak-02	Pyruvate+Oxalacetic acid	-0.0275	0.0381	0.8334		0.9765	
Peak-03	Alanine_2TMS_Major	0.0649	-0.0898	0.6192		0.9765	
Peak-04	Glycine_2TMS_Minor	-0.0092	0.0127	0.9440		0.9765	
Peak-05	2-Aminobutyric acid	0.0519	-0.0718	0.6911		0.9765	
Peak-06	Valine_2TMS_Major	0.0101	-0.0140	0.9383		0.9765	
Peak-07	Serine_2TMS_Minor	0.0469	-0.0649	0.7196		0.9765	
Peak-08	Isoleucine_2TMS	-0.1283	0.1775	0.3231		0.9765	
Peak-09	Threonine_2TMS	0.0408	-0.0564	0.7553		0.9765	
Peak-10	Proline	-0.0302	0.0418	0.8175		0.9765	
Peak-11	Glycine_3TMS_Major	0.0372	-0.0515	0.7760		0.9765	
Peak-12	Succinic acid(or aldehyde)	-0.0578	0.0799	0.6584		0.9765	
Peak-13	Fumaric acid	0.0113	-0.0157	0.9310		0.9765	
Peak-14	Serine_3TMS_Major	0.0445	-0.0615	0.7337		0.9765	
Peak-15	Alanine_3TMS_Minor	0.0674	-0.0933	0.6056		0.9765	
Peak-16	Threonine_3TMS	0.0275	-0.0380	0.8337		0.9765	
Peak-17		0.0526	-0.0728	0.6871		0.9765	
Peak-18	homoserine	0.0744	-0.1029	0.5687		0.9765	
Peak-19		0.0428	-0.0592	0.7433		0.9765	
Peak-20	Malic acid	0.0322	-0.0445	0.8056		0.9765	
Peak-21		0.0691	-0.0955	0.5970		0.9765	
Peak-23	Aspartic acid	0.0515	-0.0713	0.6934		0.9765	
Peak-24	Methionine	0.0376	-0.0520	0.7739		0.9765	
Peak-25	Pyroglutamic acid	0.0251	-0.0347	0.8479		0.9765	
Peak-26	4-Aminobutyric acid	0.0275	-0.0381	0.8333		0.9765	
Peak-27		0.0321	-0.0444	0.8061		0.9765	
Peak-28		0.0708	-0.0979	0.5878		0.9765	
Peak-29		0.0206	-0.0285	0.8750		0.9765	
Peak-30		-0.1080	0.1493	0.4068		0.9765	
Peak-31		0.0374	-0.0517	0.7750		0.9765	
Peak-32		-0.0160	0.0221	0.9028		0.9765	
Peak-33	Glutamic acid	0.0297	-0.0411	0.8204		0.9765	
Peak-34	Phenylalanine	0.0642	-0.0888	0.6233		0.9765	
Peak-36		0.0131	-0.0181	0.9204		0.9765	
Peak-38	Asparagine	0.1077	-0.1490	0.4078		0.9765	
Peak-39		0.0424	-0.0587	0.7455		0.9765	
Peak-40		0.0283	-0.0392	0.8285		0.9765	
Peak-43		0.0832	-0.1151	0.5235		0.9765	
Peak-45		0.0014	-0.0020	0.9914		0.9940	
Peak-46		0.0124	-0.0172	0.9243		0.9765	
Peak-47	Glutamine_3TMS	0.0849	-0.1174	0.5152		0.9765	
Peak-48	Citric acid + Isocitric acid	-0.1170	0.1619	0.3682		0.9765	
Peak-49	Ornithine	-0.1070	0.1480	0.4111		0.9765	
Peak-50		0.0370	-0.0512	0.7773		0.9765	
Peak-51	Lysine_3TMS_Minor	-0.3693	0.5109	0.0024	*	0.0357	*
Peak-52	Allose_1_Major	-0.1341	0.1855	0.3013		0.9765	
Peak-53		0.0304	-0.0420	0.8165		0.9765	
Peak-54	Glucose_2_Minor	0.0333	-0.0461	0.7989		0.9765	
Peak-55	Lysine_4TMS_Major	-0.3764	0.5207	0.0019	*	0.0357	*
Peak-57	Histidine	-0.5140	0.7110	0.0000	*	0.0001	*
Peak-59		0.0319	-0.0442	0.8072		0.9765	
Peak-60		0.0338	-0.0468	0.7959		0.9765	
Peak-61		0.0328	-0.0454	0.8021		0.9765	
Peak-62		0.0333	-0.0460	0.7993		0.9765	
Peak-63		-0.5163	0.7142	0.0000	*	0.0001	*
Peak-64		-0.1203	0.1665	0.3545		0.9765	
Peak-66		0.0010	-0.0014	0.9940		0.9940	
Peak-67	Trehalose	-0.0454	0.0628	0.7283		0.9765	
Peak-68	Melibiose_1_Major	0.0296	-0.0410	0.8208		0.9765	
Peak-69		-0.0486	0.0673	0.7099		0.9765	

Table S2. Statistically significantly metabolites correlated with the OS-PC1 score obtained by OS-PCA of metabolome data for taste tasting of Japanese green tea.

Metabolite Name	w_x	R	*p*-value		*q*-value	
2-Hydroxypyridine	-0.0950	-0.5730	0.0834		0.4442	
Pyruvate+Oxalacetic acid	-0.0876	-0.5285	0.1163		0.5132	
Noise1(for silyl)	-0.0269	-0.1624	0.6540		0.9140	
Alanine_2TMS_Major	0.0778	0.4691	0.1714		0.5956	
Unknown_1_Organic acid like	-0.0195	-0.1178	0.7458		0.9552	
Noise6(for silyl)	-0.0202	-0.1219	0.7372		0.9552	
n-Butylamine	-0.0303	-0.1830	0.6129		0.8917	
Oxalate	-0.0055	-0.0333	0.9273		0.9716	
Unknown_2_Organic acid like	-0.0341	-0.2056	0.5688		0.8766	
Unknown_3_Amine like	-0.0694	-0.4186	0.2286		0.6949	
Malonic acid	-0.0902	-0.5441	0.1039		0.4975	
Valine_2TMS_Major	0.0049	0.0297	0.9350		0.9716	
Unknown_4_Organic acid like	0.0081	0.0490	0.8931		0.9708	
Unknown_5_Amine like	0.0198	0.1192	0.7429		0.9552	
Urea	0.0032	0.0194	0.9575		0.9793	
Serine_2TMS_Minor	0.1317	0.7940	0.0061	*	0.1504	*
2-Aminoethanol	0.0114	0.0685	0.8510		0.9652	
Unknown_6	0.1191	0.7183	0.0193	*	0.2521	
Unknown_7	0.1181	0.7123	0.0208	*	0.2521	
Phosphate	0.1178	0.7105	0.0213	*	0.2521	
Leucine_2TMS	-0.0129	-0.0777	0.8311		0.9652	
Isoleucine_2TMS	-0.0226	-0.1364	0.7070		0.9469	
Threonine_2TMS	0.0742	0.4473	0.1949		0.6370	
Proline	0.0112	0.0674	0.8532		0.9652	
Glycine_3TMS_Major	0.1053	0.6352	0.0484	*	0.3288	
Unknown_8	0.0191	0.1151	0.7515		0.9552	
Succinic acid(or aldehyde)	0.0195	0.1179	0.7457		0.9552	
Glyceric acid	-0.0704	-0.4245	0.2214		0.6868	
Fumaric acid	0.0239	0.1442	0.6911		0.9398	
Serine_3TMS_Major	0.1164	0.7022	0.0236	*	0.2653	
Alanine_3TMS_Minor	-0.0739	-0.4454	0.1970		0.6370	
Unknwon_9	0.0824	0.4970	0.1439		0.5581	
Unknown_10	-0.1185	-0.7146	0.0202	*	0.2521	
Unknown_11_Sugar like	0.0631	0.3805	0.2781		0.7449	
Threonine_3TMS	0.0644	0.3881	0.2678		0.7449	
Unknown_12_Organic acid like	-0.0442	-0.2667	0.4564		0.8516	
Unknown_13_Organic acid like	0.0343	0.2068	0.5664		0.8766	
Unknown_14_Organic acid like	-0.0147	-0.0885	0.8080		0.9652	
b-Alanine	-0.0131	-0.0789	0.8285		0.9652	
Noise2(for silyl)	-0.0121	-0.0733	0.8406		0.9652	
Unknown_15_Organic acid like	0.1098	0.6621	0.0370	*	0.3288	
homoserine	0.1083	0.6534	0.0405	*	0.3288	
Noise3(for silyl)	-0.0296	-0.1783	0.6222		0.8917	
Citramalic acid	-0.0454	-0.2738	0.4440		0.8516	
Malic acid	-0.1062	-0.6403	0.0461	*	0.3288	
Noise4(for silyl)	-0.1383	-0.8341	0.0027	*	0.1504	*
Unknown_16_Sugar like	-0.0596	-0.3596	0.3074		0.8037	
meso-erythritol	-0.0735	-0.4430	0.1998		0.6370	
Noise5(for silyl)	-0.1309	-0.7892	0.0066	*	0.1504	*
Adipic acid	-0.1378	-0.8311	0.0029	*	0.1504	*
Unknown_17	0.0371	0.2240	0.5339		0.8766	
Aspartic acid	0.0329	0.1985	0.5825		0.8833	

Unknown_18_Organic acid like	0.0067	0.0402	0.9122		0.9716	
Methionine	0.0646	0.3893	0.2662		0.7449	
a-Phenylglycine	0.0576	0.3476	0.3251		0.8037	
trans-4-Hydroxy-L-proline	-0.0825	-0.4977	0.1432		0.5581	
Pyroglutamic acid	0.0327	0.1972	0.5849		0.8833	
4-Aminobutyric acid	-0.0540	-0.3258	0.3583		0.8123	
Unknown_19_Sugar like	0.0194	0.1168	0.7480		0.9552	
Unknown_20_Organic acid like	-0.0044	-0.0268	0.9414		0.9716	
2-Isopropylmalic acid	-0.1050	-0.6330	0.0495	*	0.3288	
Alpha-Ketoglutaric acid	-0.0777	-0.4685	0.1721		0.5956	
Unknown_21_Organic acid like	0.0841	0.5069	0.1348		0.5431	
Unknown_22_Organic acid like	-0.0127	-0.0766	0.8334		0.9652	
1-Methyl uracil	-0.0128	-0.0773	0.8320		0.9652	
Unknown_23_Organic acid like	-0.0134	-0.0808	0.8245		0.9652	
Unknown_24	0.0047	0.0283	0.9381		0.9716	
Glutamic acid	-0.0102	-0.0613	0.8665		0.9652	
Unknown_25	0.0109	0.0659	0.8564		0.9652	
Xylose_1(or Lyxose_1)_Minor	0.0888	0.5356	0.1106		0.5077	
Phenylalanine	-0.0317	-0.1912	0.5966		0.8890	
Xylose_2_Major	-0.1035	-0.6244	0.0537		0.3353	
threo-3-Hydroxy-L-aspartic acid	-0.1353	-0.8158	0.0040	*	0.1504	*
Arabinose	-0.1346	-0.8114	0.0044	*	0.1504	*
Unknown_26_Organic acid like	-0.1109	-0.6686	0.0345	*	0.3288	
Asparagine	0.0798	0.4815	0.1588		0.5764	
Unknown_27_Organic acid like	0.0036	0.0216	0.9529		0.9790	
Unknown_28_Sugar	0.0164	0.0989	0.7858		0.9652	
Unknown_29_Organic acid like	0.0639	0.3856	0.2711		0.7449	
Arabitol	-0.0059	-0.0354	0.9226		0.9716	
Unknown_30_Organic acid like	0.0092	0.0553	0.8794		0.9652	
Unknown_31	-0.0164	-0.0989	0.7858		0.9652	
Ribitol	0.0088	0.0533	0.8838		0.9653	
Unknown_32	0.0126	0.0762	0.8342		0.9652	
Unknown_33	0.0441	0.2658	0.4580		0.8516	
Unknown_34_Sugar like	0.0942	0.5678	0.0869		0.4442	
Putrescine	0.0487	0.2938	0.4100		0.8311	
Unknown_35	-0.1069	-0.6447	0.0442	*	0.3288	
Unknown_36_Organic acid like	-0.0951	-0.5735	0.0830		0.4442	
Unknown_37_Sugar like	-0.0305	-0.1838	0.6113		0.8917	
Glutathione Oxidized	-0.0262	-0.1580	0.6628		0.9159	
Theanine_1_Same_dif	-0.0422	-0.2544	0.4782		0.8527	
Unknown_38	-0.0004	-0.0024	0.9948		0.9994	
Unknown_39_Sugar like	0.0258	0.1553	0.6683		0.9168	
Glutamine_3TMS	0.0553	0.3332	0.3468		0.8123	
2-Deoxy-D-glucose	0.0421	0.2540	0.4789		0.8527	
O-Phosphoethanolamine	0.0525	0.3168	0.3725		0.8123	
Theanine_2_Same_dif	0.0844	0.5089	0.1331		0.5431	
Unknown_40_Organic acid like	-0.1241	-0.7483	0.0128	*	0.2054	*
Unknown_41	-0.1245	-0.7510	0.0123	*	0.2054	*
Shikimic acid	-0.1309	-0.7891	0.0067	*	0.1504	*
Unknown_42	-0.0451	-0.2718	0.4475		0.8516	
Citric acid + Isocitric acid	-0.0359	-0.2164	0.5482		0.8766	
Unknown_43_Organic acid like	-0.0347	-0.2091	0.5621		0.8766	
Unknown_44	-0.0108	-0.0649	0.8586		0.9652	
Ornithine	0.0170	0.1026	0.7780		0.9652	
Unknown_45_Amine like	-0.0982	-0.5920	0.0714		0.4118	
3,4-dihydroxybenzoate	-0.0971	-0.5855	0.0753		0.4238	

Citrulline	-0.0262	-0.1577	0.6635		0.9159	
Unknown_46_Organic acid like	-0.0668	-0.4031	0.2481		0.7344	
Unknown_47_Organic acid like	-0.0807	-0.4868	0.1537		0.5762	
Unknown_48_Organic acid like	0.0167	0.1009	0.7815		0.9652	
Quinic acid_like1	0.0161	0.0970	0.7898		0.9652	
Quinic acid_like2	0.0459	0.2768	0.4389		0.8516	
Unknown_49_Organic acid like	-0.0096	-0.0580	0.8735		0.9652	
Unknown_50	-0.0178	-0.1072	0.7681		0.9652	
Quinic acid_like3	0.0568	0.3424	0.3327		0.8050	
Unknown_51_Organic acid like	0.0380	0.2290	0.5244		0.8766	
Fructose_1_Major	-0.0391	-0.2357	0.5122		0.8766	
Unknown_52_Organic acid like	-0.0425	-0.2564	0.4746		0.8527	
Fructose_2_Minor	-0.0360	-0.2171	0.5468		0.8766	
Mannose_1_Major	-0.0787	-0.4747	0.1657		0.5917	
Galactose_1_Major	-0.1270	-0.7660	0.0098	*	0.1839	*
Glucose_1_Major	-0.0456	-0.2751	0.4418		0.8516	
Unknown_53	-0.0432	-0.2605	0.4672		0.8527	
Caffeine	0.1090	0.6576	0.0388	*	0.3288	
Unknown_54_Organic acid like	-0.0288	-0.1734	0.6319		0.8991	
Mannose_2(orAllose_2)_Minor	-0.0332	-0.2001	0.5793		0.8833	
Glucuronate_1_Major	-0.1046	-0.6308	0.0505		0.3288	
Galactose_2_Minor	0.0152	0.0917	0.8011		0.9652	
Unknown_55_Sugar like	0.0237	0.1431	0.6934		0.9398	
Glucose_2_Minor	-0.0547	-0.3301	0.3516		0.8123	
Unknown_56_Organic acid like	0.0634	0.3823	0.2756		0.7449	
Glucono-1,4-lactone	0.0061	0.0367	0.9198		0.9716	
Unknown_57_Organic acid like	0.0015	0.0091	0.9801		0.9933	
Unknown_58_Organic acid like	0.0147	0.0887	0.8075		0.9652	
Lysine_4TMS_Major	-0.0027	-0.0161	0.9648		0.9822	
Histidine	0.0110	0.0664	0.8553		0.9652	
Galactosamine_1_Major	0.0000	-0.0003	0.9994		0.9994	
Unknown_59_Organic acid like	0.0846	0.5103	0.1318		0.5431	
Galactitol	0.1073	0.6468	0.0433	*	0.3288	
Unknown_60_Organic acid like	0.0932	0.5621	0.0908		0.4538	
Galactosamine_2_Minor	-0.0461	-0.2779	0.4369		0.8516	
Unknown_61	0.0570	0.3438	0.3307		0.8050	
Pyridoxal	-0.0313	-0.1885	0.6021		0.8912	
Tyrosine	-0.0296	-0.1785	0.6218		0.8917	
Glucarate_Minor	-0.0494	-0.2979	0.4032		0.8311	
Gallic acid	0.0419	0.2526	0.4813		0.8527	
Unknown_62_Organic acid like	-0.0737	-0.4446	0.1980		0.6370	
Unknown_63_Sugar like	-0.0516	-0.3111	0.3815		0.8175	
Coniferyl aldehyde_1_Major	-0.0523	-0.3156	0.3744		0.8123	
Glucarate_Major	0.0390	0.2351	0.5132		0.8766	
Unknown_64_Sugar like	-0.0584	-0.3519	0.3186		0.8037	
Unknown_65_Organic acid like	0.0343	0.2070	0.5662		0.8766	
Gluconic acid	-0.1019	-0.6146	0.0587		0.3568	
Unknown_66_Organic acid like	0.0194	0.1170	0.7475		0.9552	
Unknown_67_Sugar like	-0.0733	-0.4419	0.2010		0.6370	
Unknown_68_Organic acid like	0.0300	0.1811	0.6166		0.8917	
Unknown_69	-0.0945	-0.5698	0.0855		0.4442	
Inositol	-0.0406	-0.2450	0.4950		0.8701	
Lanthionine	-0.0752	-0.4537	0.1878		0.6370	
Unknown_70_Sugar like	-0.0866	-0.5225	0.1213		0.5249	
Unknown_71	-0.0800	-0.4823	0.1580		0.5764	
Unknown_72_Organic acid like	0.0359	0.2166	0.5477		0.8766	

Unknown_73_Organic acid like	-0.0092	-0.0555	0.8789		0.9652	
Unknown_74	0.0613	0.3694	0.2935		0.7768	
Unknown_75	0.0632	0.3810	0.2774		0.7449	
Unknown_76_Sugar phosphate like	0.1044	0.6295	0.0511		0.3288	
Unknown_77_Organic acid like	0.0579	0.3490	0.3229		0.8037	
Unknown_78	-0.0372	-0.2243	0.5332		0.8766	
Unknown_79_Organic acid like	-0.0559	-0.3369	0.3411		0.8123	
Tryptophan	-0.0142	-0.0857	0.8138		0.9652	
Octadecanoate	0.0809	0.4877	0.1527		0.5762	
Unknown_80_Sugar like	-0.0195	-0.1175	0.7466		0.9552	
Unknown_81_Sugar phosphate like	0.0230	0.1385	0.7028		0.9469	
Unknown_82_Organic acid like	-0.0425	-0.2560	0.4752		0.8527	
Fructose 6-Phosphate_1	0.0285	0.1717	0.6353		0.8991	
Unknown_83_Organic acid like	0.0046	0.0276	0.9396		0.9716	
Unknown_84	-0.0109	-0.0657	0.8569		0.9652	
Fructose 6-Phosphate_2	0.0395	0.2385	0.5070		0.8766	
Unknown_85_Sugar like	0.0537	0.3236	0.3618		0.8123	
Unknown_86_Organic acid like	-0.0347	-0.2095	0.5614		0.8766	
Unknown_87_Organic acid like	-0.0505	-0.3047	0.3919		0.8280	
Unknown_88_Organic acid like	-0.1138	-0.6865	0.0283	*	0.2900	
Unknown_89_Organic acid like	-0.0050	-0.0299	0.9347		0.9716	
Unknown_90_Organic acid like	-0.0532	-0.3206	0.3664		0.8123	
Unknown_91	0.0359	0.2163	0.5483		0.8766	
Unknown_92_Sugar like	0.0323	0.1946	0.5902		0.8852	
Unknown_93	0.0591	0.3561	0.3125		0.8037	
Unknown_94_Organic acid like	0.1468	0.8852	0.0007	*	0.0744	*
Unknown_95	0.1138	0.6865	0.0284	*	0.2900	
Sucrose	0.0529	0.3190	0.3690		0.8123	
Unknown_96_Organic acid like	0.0372	0.2242	0.5334		0.8766	
Unknown_97_Sugar like	0.1308	0.7888	0.0067	*	0.1504	*
Unknown_98_Sugar like	-0.0634	-0.3825	0.2754		0.7449	
Unknown_99	-0.0468	-0.2824	0.4291		0.8516	
Unknown_100_Organic acid like	-0.1003	-0.6046	0.0641		0.3795	
b-Lactose_1_Major	-0.0881	-0.5311	0.1142		0.5132	
Maltose_1_Major	-0.0840	-0.5065	0.1352		0.5431	
Trehalose	-0.0353	-0.2129	0.5548		0.8766	
Maltose_2_Minor	-0.0051	-0.0310	0.9322		0.9716	
Unknown_101_Organic acid like	-0.0444	-0.2677	0.4545		0.8516	
Melibiose_1_Major	0.0362	0.2184	0.5445		0.8766	
Unknown_102	0.0101	0.0611	0.8668		0.9652	
Unknown_103	0.0049	0.0297	0.9350		0.9716	
Epicatechin	0.1046	0.6305	0.0507		0.3288	
Melibiose_2_Minor	0.1192	0.7189	0.0191	*	0.2521	
Cyanine	0.0631	0.3806	0.2779		0.7449	
Epigallo catechin	0.0892	0.5377	0.1089		0.5077	
Epigallo catechin_like	-0.0273	-0.1649	0.6490		0.9126	
Unknown_104_Organic acid like	-0.0924	-0.5574	0.0941		0.4604	
Unknown_105_Sugar like	0.0584	0.3524	0.3179		0.8037	
Kaempferol	-0.0499	-0.3008	0.3984		0.8300	
Unknown_106_Organic acid like	0.0192	0.1156	0.7505		0.9552	
Unknown_107	-0.0002	-0.0012	0.9974		0.9994	
Unknown_108	0.0681	0.4105	0.2387		0.7160	
Unknown_109_Organic acid like	0.0702	0.4233	0.2228		0.6868	
Unknown_110	-0.0503	-0.3036	0.3938		0.8280	
Unknown_111	-0.0522	-0.3149	0.3755		0.8123	
Unknown_112	0.1083	0.6530	0.0406	*	0.3288	

Unknown_113_Organic acid like	-0.0443	-0.2670	0.4559		0.8516	
Unknown_114_Organic acid like	0.0488	0.2944	0.4090		0.8311	
Raffinose	-0.1566	-0.9445	0.0000	*	0.0087	*
Unknown_115_Organic acid like	-0.1270	-0.7658	0.0098	*	0.1839	*
Unknown_116_Sugar phosphate like	-0.0540	-0.3255	0.3588		0.8123	
